# VP0 Myristoylation Is Essential for Senecavirus A Replication

**DOI:** 10.3390/pathogens13070601

**Published:** 2024-07-21

**Authors:** Peiyu Xiao, Liang Meng, Xingyang Cui, Xinran Liu, Lei Qin, Fandan Meng, Xuehui Cai, Dongni Kong, Tongqing An, Haiwei Wang

**Affiliations:** 1 State Key Laboratory of Veterinary Biotechnology, Harbin Veterinary Research Institute, Chinese Academy of Agricultural Sciences, Harbin 150069, China; matchxpy@126.com (P.X.); mengl98@163.com (L.M.); cxy_2010@126.com (X.C.); qinlei-@163.com (L.Q.); mengfandan@caas.cn (F.M.); caixuehui@caas.cn (X.C.); 2 Regeneron Pharmaceuticals Inc., 777 Old Saw Mill River Road, Tarrytown, New York, NY 10591, USA; xinran.liu@regeneron.com; 3 Heilongjiang Provincial Research Center for Veterinary Biomedicine, Harbin 150069, China; 4 Institute of Veterinary Drug Control, No. 8 Nandajie, Zhongguancun, Haidian, Beijing 100081, China; kongdongni@163.com; 5 Heilongjiang Provincial Key Laboratory of Veterinary Immunology, Harbin 150069, China

**Keywords:** VP0 myristoylation, virions assembly, virus replication, NMT1

## Abstract

Many picornaviruses require the myristoylation of capsid proteins for viral replication. Myristoylation is a site-specific lipidation to the N-terminal G residue of viral proteins, which is catalyzed by the ubiquitous eukaryotic enzyme N-myristoyltransferase (NMT) by allocating the myristoyl group to the N-terminal G residue. IMP-1088 and DDD85646 are two inhibitors that can deprive NMT biological functions. Whether Senecavirus A (SVA) uses NMT to modify VP0 and regulate viral replication remains unclear. Here, we found that NMT inhibitors could inhibit SVA replication. NMT1 knock-out in BHK-21 cells significantly suppressed viral replication. In contrast, the overexpression of NMT1 in BHK-21 cells benefited viral replication. These results indicated that VP0 is a potential NMT1 substrate. Moreover, we found that the myristoylation of SVA VP0 was correlated to the subcellular distribution of this protein in the cytoplasm. Further, we evaluated which residues at the N-terminus of VP0 are essential for viral replication. The substitution of N-terminal G residue, the myristoylation site of VP0, produced a nonviable virus. The T residue at the fifth position of the substrates facilitates the binding of the substrates to NMT. And our results showed that the T residue at the fifth position of VP0 played a positive role in SVA replication. Taken together, we demonstrated that SVA VP0 myristoylation plays an essential role in SVA replication.

## 1. Introduction

Senecavirus A (SVA) is one of the causative agents of vesicular diseases in swine [1,2]. SVA is a non-enveloped, positive-sense, single-stranded RNA virus in the genus *Senecavirus*, a member of *Picornaviridae* family [3]. In a mature virus, the SVA genome is encapsulated into a viral capsid, composed of 60 asymmetric units, with each made of VP0, VP3, and VP1. The genome contains a single open reading frame (ORF), which translates into a polyprotein, including capsid precursor protein (P1) and nonstructural precursor proteins (P2 and P3) [3,4]. The P1 protein is processed by a virus-encoded protease to produce three capsid proteins VP0, VP1, and VP3 [5]. VP0 in the viral particle can be further cleaved into VP2 and VP4 [6]. During translation, assembly, and packaging in host cells, viral capsids may undergo a series of modifications regulated by cellular enzymes to increase functional diversity. Lipidation is an important co- or post-translational modification in cell signaling pathways and functional proteins’ regulation to mediate protein conformation, stability, and localization; target the protein to the cell membrane; and facilitate their subcellular trafficking [7,8]. The lipidation of viral proteins was found to partially contribute to viral pathogenesis. For example, myristoylation, a common lipidation strategy, has been leveraged to acquire proper functions for many viral proteins, including the Gag protein of the human immunodeficient virus, the Z protein of the Lassa virus, and matrix protein of the spleen necrosis virus [9,10,11].

Myristoylation is a fatty acylation process in eukaryotes, catalyzed by N-myristoyltransferase (NMT) with myristoyl-CoA as co-substrate and source of the myristoyl group [12]. This process covalently attaches the myristoyl group to the alpha-amino group of an N-terminal G residue over a wide range of substrate proteins [13]. Due to improved binding affinity to the cell membrane, many cellular proteins are myristoylated to switch localization and regulate signaling pathways [7,8]. Myristoylation occurs in various ways. It can co-translationally modify newly synthesized peptides after cleavage of the initial amino acid methionine (Met) by aminopeptidase 2 (MetAP2) [14]. It can also post-translationally modify internal G residue exposed outside. Myristoylation favors a consensus sequence of G^1^X^2^X^3^X^4^S/T^5^X^6^X^7^X^8^ [15]. Previous studies have explored the use of this consensus motif to myristoylate viral proteins, which play essential roles in many stages of the viral lifecycle. For example, myristoylated capsid protein VP4 was reported in many picornaviruses, including bovine enterovirus, poliovirus, Foot and Mouth Disease Virus, and rhinovirus (RV) [16]. In studies involving coxsackievirus B3 and enterovirus 71, myristoylated VP4 protein is required in the viral replication [17,18]. Myristoylation of human rhinovirus N-terminal VP4 protein has been proven to contribute to membrane pore-forming activity, enhance membrane permeability, and facilitate the viral genome transfer into the cytoplasm [19,20]. In another study involving RV, myristoylated VP4 was shown as a ligand that activates TLR2-dependent proinflammatory responses [21]. These results suggested that the myristoylation of viral protein is closely related to viral biology, pathogenesis, and host innate immune response. 

In this study, we explored myristoylation at capsid protein VP0 and the roles of myristoylated VP0 in SVA replication. The results showed that SVA VP0 subcellular localization was regulated by its myristoylation and SVA replication could be suppressed when NMT activity was inhibited or the NMT1 expression level declined sharply in BHK-21 cells. Our results could be a reference for anti-virus medicine and SVA attenuated vaccine development. 

## 2. Materials and Methods

### 2.1. Cell Lines and Viruses

BHK-21 cells and ST cells were grown in Dulbecco’s modified Eagle medium (DMEM) (Gibco, Grand Island, NY, USA) containing 10% fetal bovine serum (FBS) at 37 °C and 5% CO_2_. The wild-type SVA/HLJ/CHA/2016 was described previously [22]. The pSVA-eGFP and pSVA-WT infectious clones were constructed in our previous work, and SVA-eGFP was rescued [22].

### 2.2. Plasmid Construction 

The NMT1 gene from BHK-21 cells was amplified by using primers (NMT1-*Nhe*I-F and NMT1-*Not*I-R). The amplified NMT1 gene and vector pCI-tb206-2 kept in our lab was digested by *Nhe*I and *Not*I to generate linear DNA. These two digested DNAs were linked by T4 ligase (ThermoFisher, USA). The SVA wild-type VP0, VP0-G1A, and eGFP gene were amplified by using corresponding primers (VP0-*Nhe*I-F, VP0-G1A-*Nhe*I-F, VP0-linker-R, eGFP-linker-F, and eGFP-*Not*I-R). VP0 and its mutant VP0-G1A, fused with an eGFP sequence by an (EKKKR)2 linker using the in-fusion technique, were cloned into pCI-tb206-2, respectively. These two constructed plasmids were named pVP0-WT-eGFP and pVP0-G1A-eGFP. The pSVA-eGFP-VP0-G1A/K containing SVA-eGFP VP0 mutations was constructed based on infectious clone pSVA-eGFP, and the pSVA-VP0-T5A/K containing SVA VP0 mutations was constructed based on the SVA infectious clone pSVA16 (pSVA-WT) [22,23]. VP0 and its mutant gene were amplified by using corresponding primers (VP0-*Nhe*I-F, VP0-G1A-*Sac*II-R, VP0-G1K-*Sac*II-R, VP0-T5A-*Sac*II-R, and VP0-T5K-*Sac*II-R). Infectious clones, the amplified VP0 gene, and its mutans gene were digested by *Nhe*I and *Sac*II. VP0 and its mutant VP0-G1A/K and VP0-T5A/K were cloned into pSVA16 or pSVA-eGFP, respectively. Primers used in plasmid construction are listed in Table S1.

### 2.3. Recovery of Mutant Viruses

BHK-21 cells seeded in a 6-well plate with 60~70% confluency were transfected with the pSVA infectious clone or mutants with or without eGFP using Lipofectamine 3000 reagent (Life Technologies, Carlsbad, CA, USA). The mixture of clones and Lipofectamine 3000 was added to BHK-21 cells and incubated for 6 h. The culture medium in the 6-well plate was then replaced by DMEM supplemented with 2% FBS. At 48 h post-transfection, virus stocks were subsequently made in BHK-21 cells for further analysis.

### 2.4. Preparation of BHK-21 Derived Cell Lines with Knock-Out or Overexpression of NMT1

The sgRNAs targeting NMT1 exons were designed and analyzed with CRISPOR. Designed sgRNAs were listed in Table S2.

Oligos were diluted at a concentration of 100 μM. An amount of 1 μL each of the sense and antisense primers were added to a mixture of 6.5 μL ddH_2_O, 0.5 μL T4 PNK (Polynucleotide kinase), and 1 μL T4 PNK buffer and each pair of oligos was annealed under a program [37 °C 30 min, 95 °C 5 min, and then ramp down to 25 °C at 5 °C/min]. An amount of 1 μL annealed oligos, 100 ng uncut lentiCRISPR v2 GFP (Addgene: #82416), 0.5 μL *Esp*3I, 1 μL buffer (10×) for *Esp*3I, 0.6 μL T4 ligase (NEB, Ipswich, MA, USA), and 1 μL buffer (10×) for T4 ligase were mixed with 5.9 μL ddH_2_O. This mixture was incubated at 2 temperature values [37 °C for 5 min, 16 °C for 10 min], the reaction was repeated 50 times, then it was incubated at 37 °C for 15 min and 80 °C for 5 min. The mixture was cooled down to 4 °C after the reaction. BHK-21 cells were then transfected with the sgRNA fused lentiCRISPR v2 GFP to generate NMT1-knock-out (KO) cells and were sorted by flow cytometry. Western blot and DNA sequencing confirmed the sorted BHK-21 NMT1 knock-out cells (BHK-NTM1-KO).

To overexpress the NMT1 in BHK-21 cells (BHK-NMT1), BHK-21 cells were seeded in a 6-well plate until the cell monolayer reached 50~60% confluency and was transfected with pCI-NMT1 expressing NMT1 as described above, following the manufacturer’s instructions, and incubated for 24 h prior to viral infection.

### 2.5. Replication of SVA-eGFP or SVA-eGFP Mutant Viruses

SVA-eGFP viruses were constructed and rescued as described above [22,23]. BHK-21 or ST cells were treated with IMP-1088 (Cayman, Ann Arbor, MI, USA) or DDD85646 (Cayman, Ann Arbor, MI, USA) at the indicated concentration, respectively, or mock-treated with 0.5% DMSO for 2 h. Untreated/treated ST, BHK-21, or NMT1-modulating BHK-21-derived cell monolayers in 6-well tissue culture plates were washed with PBS and inoculated with SVA-eGFP or SVA-eGFP mutant viruses at the indicated multiplicity of infection (MOI). The plates were incubated for 1 h at 37 °C. The infected cells were then washed with PBS three times and cultured with DMEM containing 2% FBS with or without IMP-1088 or DDD85646. The cells were then fixed with 4% paraformaldehyde and permeabilized with 0.1% Triton-X 100. After washing with PBS, samples were visualized with a Leica SP5 confocal system.

pSVA-eGFP and pSVA-eGFP-VP0-G1A/K were transfected into BHK-21 cells. After 48 h transfection, the supernatants were collected. Then the same volumes of supernatants were used to infect BHK-21 cells.

### 2.6. TCID_50_ Assay and Virus Growth Curves

Ten-fold serial dilutions of viruses were prepared in 96-well round-bottom plates in DMEM and 50 μL of the dilution was transferred to 10^4^ BHK-21 cells plated in 100 μL of DMEM with 2% FBS. TCID_50_ values were determined by the Reed–Muench formula.

Viral replication kinetics was performed as follows. BHK-21 cells were treated with IMP-1088 (100 nM) or DMSO (0.5%) for 2 h. Untreated/treated BHK-21 or NMT1-modulating BHK-21-derived cell monolayers in 6-well tissue culture plates were washed with PBS, then DMEM supplemented with 2% FBS was re-added with a corresponding dose of DMSO or IMP-1088, inoculated with SVA-WT or SVA VP0 mutant viruses at the indicated MOI. The doses of DMSO, IMP-1088, and DDD85646 were the same. The plates were incubated for 1 h at 37 °C. The infected cells were then washed three times with PBS to remove unbound virus particles and covered with DMEM containing 2% FBS with or without IMP-1088. The infected cells were incubated at 37 °C and harvested at indicated times. Then, the viral titers were determined by TCID_50_ assay.

### 2.7. Localization of VP0-eGFP and VP0-G1A-eGFP in BHK-21 Cells or BHK-21 Derived Cells

BHK-21 cells with 60~70% confluency was pre-treated with IMP-1088 (100 nM) or DDD85646 (6 μM) for 2 h and transfected with either pVP0-WT-eGFP or pVP0-G1A-eGFP using Lipofectamine 3000 reagent. BHK-NMT1-KO cells grew to about 60~70% confluency and were then transfected with pVP0-WT-eGFP using the Lipofectamine 3000 reagent. The mixture of clones and Lipofectamine 3000 was added to BHK-21 cells and incubated for 24 h. The cells were then fixed with 4% paraformaldehyde and permeabilized with 0.1% Triton-X 100. After washing with PBS, the samples were visualized with the Leica SP5 confocal system or an inverted fluorescent microscope.

### 2.8. Western Blot

Cell lysates were loaded and separated under denaturing conditions in a 12% SDS-PAGE. After transfer onto a polyvinylidene difluoride (PVDF) membrane, the PVDF was blocked with 5% skim milk in PBS at room temperature, followed by an additional incubation of 1 h with an anti-NMT1 polyclonal antibody purchased from ATLAS. Then, the PVDF was washed with PBS-T three times. Mouse horseradish peroxidase-coupled monoclonal antibody specific to GAPDH and purchased from Alphabio was used as a loading control. The secondary LI-COR (IRDye® 800 CW) goat anti-rabbit IgG and IRDye® 800 CW goat anti-mouse IgG antibodies were used to detect the signal using a near-infrared fluorescence scanning imaging system (Licor Odyssey, Lincoln, NE, USA). At least three independent experiments were performed.

### 2.9. Viral Plaque Assay

Ten-fold serial dilutions of SVA-WT and SVA-VP0-T5A/K were made by serially adding 100 μL of wild-type or mutant virus to 900 μL DMEM supplemented with 2% FBS. The diluted viruses were added to ST cells seeded in a 6-well plate with 100% confluency and incubated for 1 h in 37 °C and 5% CO_2_. The culture media were then replaced by DMEM supplemented with 2% FBS and 5% methylcellulose. DMEM and methylcellulose in the plate were removed when plaques were observed. The cells were washed with PBS and stained with crystal violet.

### 2.10. Statistical Analysis

All data were obtained from three independent assays, and results were described with mean and ± standard deviations (SDs). Images were analyzed with ImageJ software (1.54j). Data were analyzed by one-way ANOVA using the GraphPad 8.0 software. All the reported *p* values were 2-sided and when *p* < 0.05 was considered statistically significant.

## 3. Results

### 3.1. NMT Inhibitors Reduced SVA Replication

**Figure 1:**
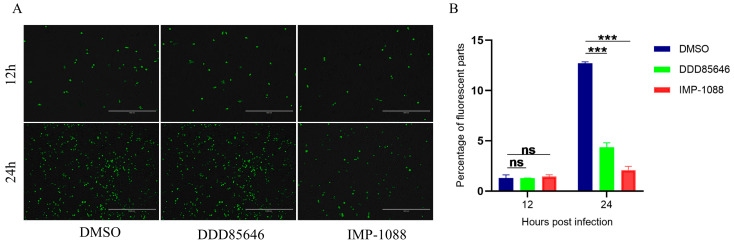
Effect of DDD85646 and IMP-1088 on SVA replication. (**A**) BHK-21 cells were infected with SVA-eGFP at an MOI of 0.1 in the absence (DMSO control) or presence of 6 μM DDD85646 and 100 nM IMP-1088 and observed under a fluorescent microscope at 12 hpi and 24 hpi. (**B**) Percentage of fluorescent parts from ImageJ analysis in (**A**). Data presented as mean ± SD of three independent experiments, *** *p* < 0.001.

Previous studies have shown that DDD85646 and IMP-1088 suppressed the NMT activity of cells [18,24,25]. Among these, IMP-1088 inhibited NMT with higher specificity and less host toxicity [24]. To investigate whether NMT1 affects SVA replication, SVA-eGFP viruses were analyzed in BHK-21 cells in the absence (DMSO control) or presence of DDD85646 (6 μM) or IMP-1088 (100 nM). According to the fluorescent intensity, approximately equal amounts of cells were observed at 12 h post infection (hpi) in all conditions (Figure 1A). However, fewer cells were infected in the presence of DDD85646 or IMP-1088 and the percentage of infected cells in the inhibitor-treated condition was also significantly lower than in DMSO-treated control condition at 24 hpi (Figure 1A,B). These results indicated that both NMT inhibitors negatively impacted SVA replication at the late stage of SVA infection.

### 3.2. NMT Inhibitors Suppressed SVA Replication in a Dose-Dependent Manner and Altered VP0 Localization

**Figure 2:**
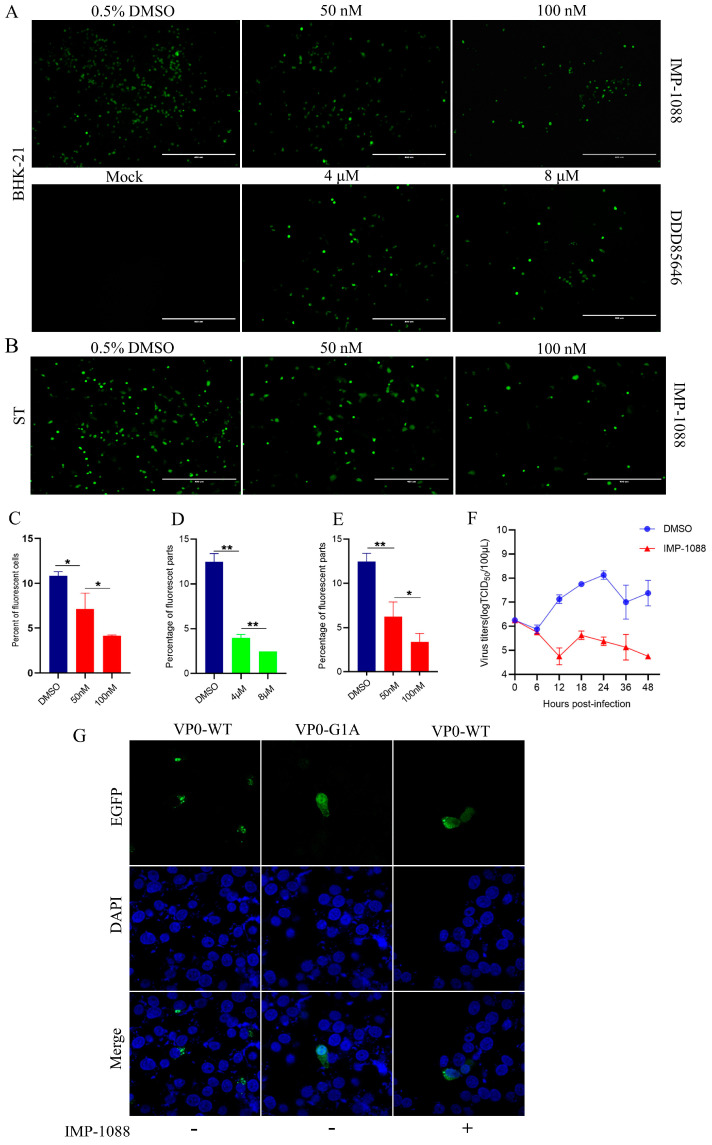
DDD85646 and IMP-1088 disturbed SVA-eGFP replication in a dose-dependent manner. (**A**) BHK-21 cells were infected with SVA-eGFP at an MOI of 0.1 in the absence (DMSO control) or presence of 50 nM and 100 nM IMP-1088 and 4 μM and 8 μM DDD85646 and observed under a fluorescent microscope at 12 hpi and 24 hpi. (**B**) ST cells were infected with SVA-eGFP at an MOI of 0.1 in the absence (DMSO control) or presence of 50 nM and 100 nM IMP-1088. (**C**,**D**) show the percentage of fluorescence levels from ImageJ analysis in (**A**). (**E**) Percentage of fluorescence levels from ImageJ analysis in (**B**). (**F**) BHK-21 cells were treated with DMSO and IMP-1088 at 0.5% and 100 nM concentrations, respectively, and then infected with SVA-WT at an MOI of 1. Supernatants, including cell lysates, were collected at indicated time points for TCID_50_ assay. Data are presented as mean ± SD of three independent experiments, 0.01 < * *p* < 0.05, 0.001 < ** *p* < 0.01. (**G**) The influence of myristoylation on VP0 subcellular localization. pVP0-WT-eGFP was transfected into BHK-21 cells in the absence or presence of IMP-1088. pVP0-G1A- eGFP was transfected into BHK-21 cells. After 24 h transfection, the subcellular localization of VP0-WT-eGFP and VP0-G1A-eGFP was visualized by a confocal microscope.

We evaluated the ability of SVA-eGFP replication in BHK-21 and ST cells with different concentrations of NMT inhibitors. At an MOI of 0.1, the number of infected cells was decreased with an increase in the concentration of IMP-1088 or DDD85646, and regardless of the concentrations of NMT inhibitors, the number of infected cells decreased compared to the control (DMSO) condition at 24 hpi (Figure 2A,B). In addition, the inhibitory effects on the percentage of SVA-infected cells by both NMT inhibitors showed a dose-dependent but cell-independent manner (Figure 2C–E). These results suggested that the infectivity of SVA is directly correlated with cellular myristoylation activity. We further evaluated the kinetics of SVA replication in BHK-21 cells treated with low cytotoxic IMP-1088 or DMSO. Interestingly, no increase in infectious virions was observed in the presence of IMP-1088 (Figure 2F), suggesting that NMT activity played an essential role in viral replication.

To investigate whether VP0 of SVA was myristoylated and had any effects on VP0 subcellular localization, we substituted the N-terminal G residue of SVA VP0 to A to shut down myristoylation. We initially tried to restore the infectious virion production of the SVA-VP0-G1A-eGFP mutant virus with a supply of VP0-WT proteins (data not shown). However, the mutant virus was not able to be rescued, suggesting that myristoylated VP0-WT protein alone could not replace the mutant VP0 protein to re-form infectious virions. We next examined whether VP0-WT and VP0-G1A proteins could be colocalized. Clones pVP0-WT-eGFP and pVP0-G1A-eGFP were transfected into BHK-21 cells with or without IMP-1088 pre-treatment. As shown in Figure 2G, nearly all wide-type VP0 proteins were concentrated in the cytoplasm, as observed by extremely bright fluorescence. In contrast to VP0-WT-eGFP, the myristoylation-inactivated VP0 protein VP0-G1A-eGFP was localized in both the nucleus and cytoplasm of BHK-21 cells. When BHK-21 cells were treated with IMP-1088, VP0-WT-eGFP proteins were re-distributed in the nucleus and cytoplasm, with a pattern similar to what was observed with VP0-G1A-eGFP proteins. This phenomenon proved that SVA VP0 could be localized to a specific subcellular domain and that myristoylation regulated its localization.

### 3.3. NMT1 Affected SVA Replication Efficiency

**Figure 3:**
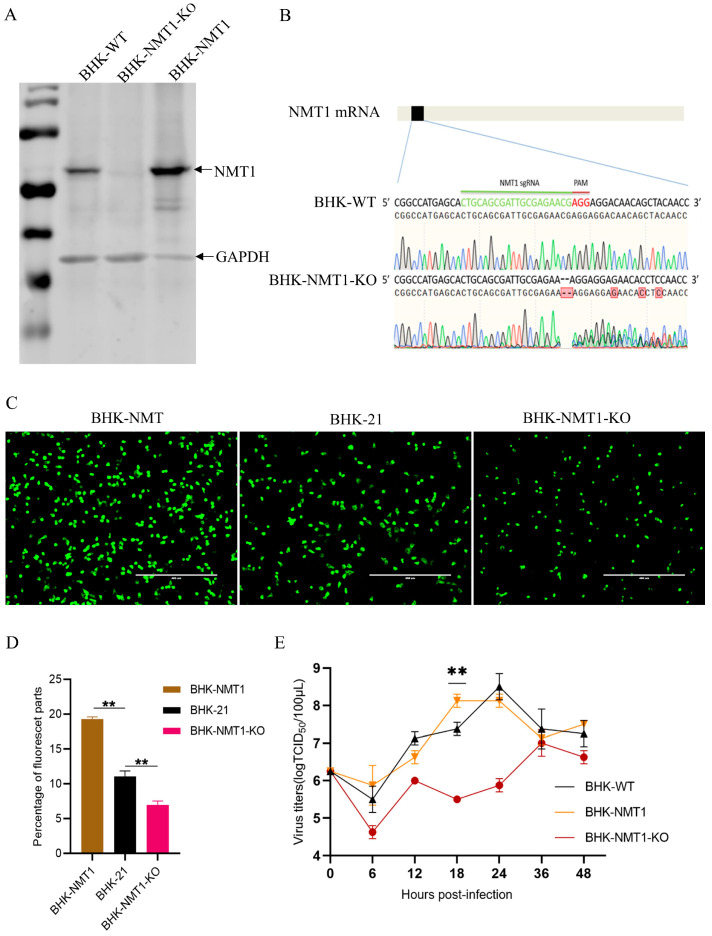
Over-expressed NMT1 in BHK-21 promoted SVA replication. (**A**) The expression of the NMT1 protein was detected in BHK-21, BHK-NMT1-KO, and BHK-NMT1 cell lines by Western blotting (WB). (**B**) The NMT1 gene of the BHK-NMT1-KO cell line was sequenced and analyzed. BHK-21 NMT1 DNA was cleaved at the sgRNA targeted site. (**C**) BHK-21, BHK-NMT1-KO, and NMT1-overexpressed BHK cells were infected with SVA-eGFP at an MOI of 0.1 and observed under a fluorescent microscope at 18 hpi. (**D**) Percentage of fluorescence levels from ImageJ analysis in (**C**). (**E**) BHK-21, BHK-NMT1-KO, and NMT1-overexpressed BHK cells were infected with SVA at an MOI of 1. Supernatants, including cells, were collected at indicated time points for the TCID_50_ assay. Data are presented as mean ± SD of three independent experiments, 0.001 < ** *p* < 0.01.

We directly examined the correlation of NMT1 activity with SVA replication using BHK-21 cells, the NMT1-knock-out BHK-21 (BHK-NMT1-KO) cell line, and NMT1-overexpression cells (BHK-NMT1). BHK-NMT1-KO was developed using sgRNAs targeting exon1/2 of NMT1 with the CRISPR/Cas9 vector lenti-v2. Western blotting analysis showed that NMT1 was successfully knocked out (Figure 3A). The NMT1 DNA sequence from BHK-NMT1-KO confirmed that the two nucleic acids were deleted at the sgRNA target site, resulting in frameshift mutation (Figure 3B). BHK-21, BHK-NMT1-KO, and BHK-NMT1 cells were infected with SVA-eGFP at an MOI of 0.1. Results showed that SVA-eGFP infected fewer BHK-NMT1-KO cells, while it infected more BHK-NMT1 cells at 18 hpi (Figure 3C,D). The amount of infected BHK-21 cells was between infected BHK-NMT1-KO and BHK-NMT1 cells at 18 hpi (Figure 3C,D). Further growth kinetic analysis showed that SVA-WT replicates much slower in BHK-NMT1-KO cells than in BHK-21 and BHK-NMT1 cells. SVA-WT replication in BHK-NMT1 cells peaked at 18 hpi, faster than BHK-21 cells at 24 hpi. Viral replication in NMT1-KO resulted in a 2.5-log decrease at 24 hpi with a peak reached at 36 hpi, compared to that in BHK-21 cells (Figure 3E). This indicated that SVA replication efficiency was associated with VP0 myristoylation. Furthermore, we observed a similar phenomenon to the above, in which VP0 localization was directly impacted by NMT. In BHK-NMT1-KO cells, fewer VP0 proteins were gathered compared to BHK-21 cells. In comparison, VP0 proteins did not gather in the subcellular domain when the first G residue of VP0 was substituted by A residue (Figure S1).

### 3.4. N-Terminal G Substitutions in VP0 Were Lethal for SVA

**Figure 4:**
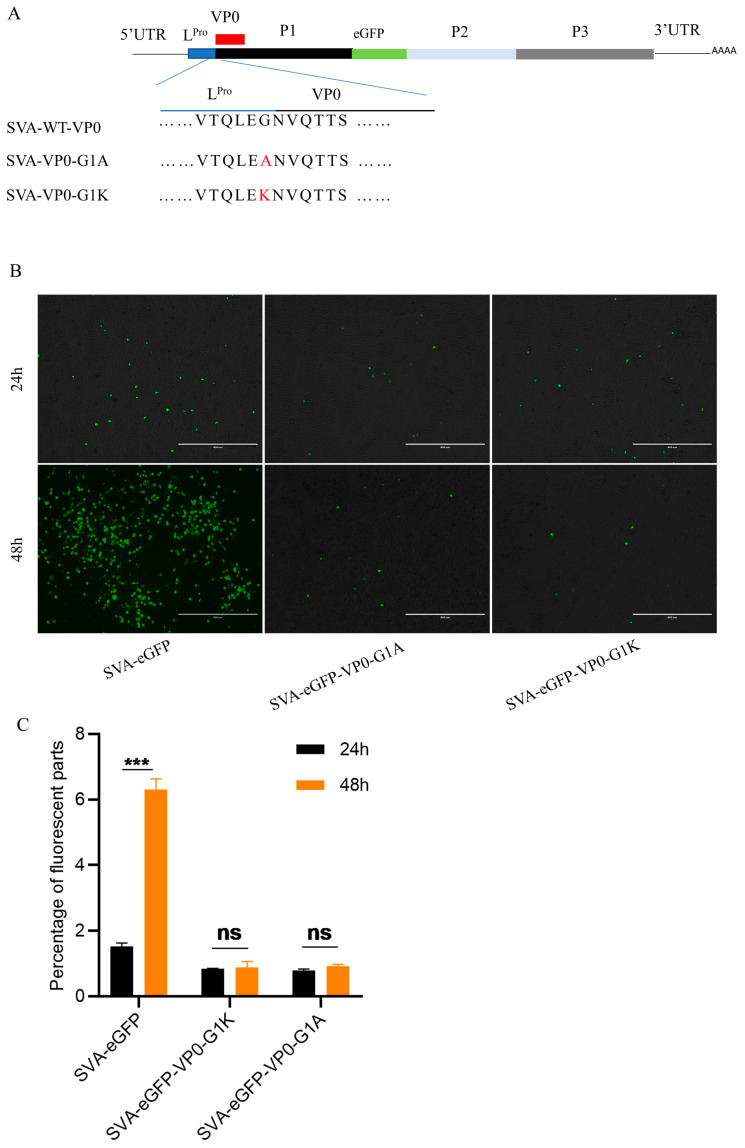
Modification of the myristoylation site of VP0 was lethal for SVA-eGFP. (**A**) The sketch map of SVA-VP0-G1A/K. (**B**) Infectious clones pSVA-eGFP-VP0-G1A/K were transfected in BHK-21. Fluorescence expression was observed at 24 h and 48 h post-transfection under a fluorescent microscope. (**C**) The fluorescence levels were analyzed by ImageJ. Data are presented as mean ± SD of three independent experiments, *** *p* < 0.001.

Our results suggested that myristoylation was essential for SVA infection. Protein VP0-G1A could not be myristoylated as our results show above. We studied what impacts on SVA replication would be produced if N-terminal G residue was substituted with A residue. pSVA-eGFP-VP0-G1A was constructed and transfected into BHK-21 cells (Figure 4A,B). At 24 h post-transfection, the green fluorescent protein was expressed in the SVA-eGFP transfected cell, and more fluorescence was detected 48 h post-transfection. However, the BHK-21 cells transfected with mutant clones showed limited green fluorescent protein expression and did not increase at 48 h post-transfection (Figure 4B,C). Apparently, SVA replication without myristoylated VP0 could not be conducted. Besides G residue, K residue is the other amino acid that could be myristoylated, according to previous reports, although in rare cases [26,27]. It would be interesting to see if replacing the myristoylation site G residue with K residue would still result in myristoylation. We supposed that SVA replication might be conducted if VP0-G1K was myristoylated. Similarly, pSVA-eGFP-VP0-G1K was constructed and transfected into BHK-21 cells (Figure 4A,B). However, we observed similar results with transfecting pSVA-eGFP-VP0-G1A. Next, the supernatants of BHK-21 transfected with pSVA-eGFP or pSVA-eGFP-G1A/K were collected and used to reinfect BHK-21 cells. At 24 hpi, CPE and green fluorescence were not observed in cells reinfected with SVA-eGFP-G1A/K recombinant viruses even at 48 hpi (data not shown). These results conclude that substitutions of the A/K residue at the N-terminus of VP0 are lethal for SVA.

### 3.5. The Residue T at the Fifth Position of VP0 Played a Positive Role in SVA Replication

**Figure 5:**
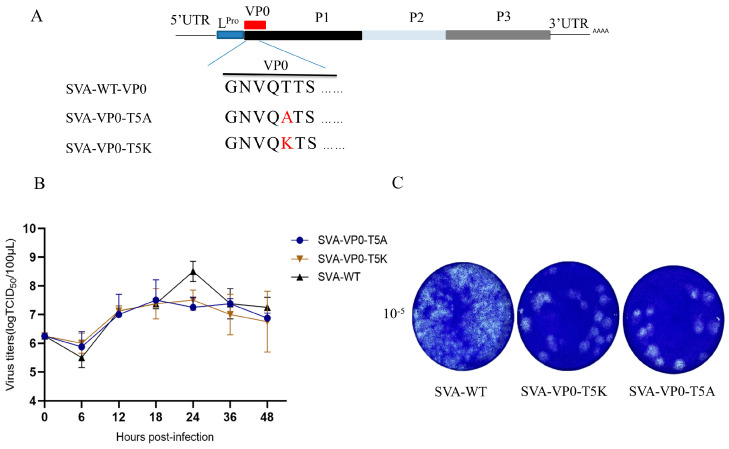
Changing the consensus amino acid sequence of NMT1 substrates declined the titer of SVA. (**A**) The sketch map of SVA-VP0-T5A/K. (**B**) Multiple-step growth curve of SVA-WT and SVA-VP0-T5A/K. BHK cells were infected with SVA-WT, SVA-VP0-T5A, or SVA-VP0-T5K at an MOI of 1. The samples were collected at indicated time points for TCID_50_ analysis. Data are presented as mean ± SD of three independent experiments, 0.001 < ** *p* < 0.01. (**C**) The replication abilities of SVA-WT, SVA-VP0-T5A, and SVA-VP0-T5K were analyzed by plaque assay on ST cells.

Many studies indicated that the fifth amino acid in the consensus sequence G^1^X^2^X^3^X^4^S/T^5^X^6^X^7^ of NMT protein substrates favors the process of myristoylation [28]. We assumed that substituting this residue would have similar effects as the N-terminal G residue substitutions. Threonine at the fifth amino acid of the SVA VP0 protein was substituted with A or K residue (Figure 5A). These two mutants, SVA-VP0-T5A and SVA-VP0-T5K, produced CPE in BHK-21 cells, and then they were further used for the multiple-step growth curve investigation. The titers of these two mutant viruses were lower than those of SVA-WT at 24 hpi (Figure 5B). And these two mutants reached a replication peak at 18 hpi, compared to SVA-WT which peaked at 24 hpi (Figure 5B). In addition, the plaque assays for SVA-WT, SVA-VP0-T5K, and SVA-VP0-T5A showed fewer plaques in the mutant-infected ST cells (Figure 5C). Therefore, VP0 still could be myristoylated when A or K residue substituted the T residue at fifth position. However, A or K residue seems to be the disadvantage of VP0 myristoylation because the mutants had lower maximum replication titers and formed fewer plaques.

## 4. Discussion

Myristoylation of capsid protein VP0 had been observed in several picornaviruses [21,29,30], implying that myristoylation is a common feature of these viruses. Previous studies have shown that VP0 myristoylation is correlated with viral infectivity and assembly [17,18,31,32]. In this study, we found that the replication of SVA was inhibited by NMT1 inhibitors IMP-1088 and DDD85646 as well as in BHK-NMT1-KO cells (Figure 1A, Figure 2A,B,F and Figure 3C,E). The number of infected BHK-21 cells treated with 50 nM IMP-1088 declined at least 4% on average compared to BHK-21 cells treated with DMSO. With the increase in the IMP-1088 dose, infected BHK-21 cells declined at least 7% (Figure 2C). A similar phenomenon was observed on ST cells. Infected ST cells treated with 50 nM or 100 nM declined approximated 5% or 8%, respectively, compared to ST cells treated with DMSO (Figure 2D). DDD85646 also hampered SVA-eGFP to infect BHK-21 cell. There was an approximately 8% or 10% decline in infected cells when BHK-21 cells were treated with 4 μM or 8μM DDD85646. This was due to the dislocation of SVA VP0 when NMT activity was contained (Figure 2G). These results confirmed that myristoylation played a significant role in SVA replication.

Myristoylation is often associated with regulating the subcellular localization of its substrate proteins as a result of facilitating their binding affinity to endomembrane and plasma membrane systems [12,33]. Depending on the localization of the modified protein substrates, it can achieve diverse functions [12]. The myristoylation-dependent localization of viral structure proteins to targeted organelles had been reported to be essential for viral assembly. For example, assembling HIV-1 and HTLV-1 virions was initiated by targeting myristoylated Gag polyprotein to the plasma membrane [34,35]. In another example, inducing the self-assembly of the FMDV pentamer occurred in a myristoylation-dependent manner [36]. Our study showed that SVA without VP0 myristoylation produced no infectious virions, suggesting that myristoylation-regulated VP0 concentration on the subcellular domain was probably indispensable for optimal viral assembly (Figure 4B).

The overexpression of NMT1 in BHK-21 cells promoted the replication of SVA and reached a peak faster than in control BHK-21 cells, but the maximum replication titer did not increase, suggesting that NMT1 regulates SVA replication efficiency (Figure 3C,E). In eukaryotes, both NMT1 and NMT2 share similar substrates with non-redundant functions. When knocking out NMT1 in BHK-21 cells, NMT2 could only partially compensate for the function of NMT1 [14]. This might be the reason for fewer myristoylated VP0 proteins gathered in the subcellular domain (Figure S1). Moreover, we found that SVA mutant viruses carrying myristoylation-deficient substitutions at the position one G residue were lethal (Figure 4B). The substitution of N-terminal G residue, adjacent to the C-terminal of L^Pro^, might affect protein cleavage efficiency between L^Pro^ and VP0. The VP0 dislocation could also impair the virion assembly due to lacking myristoyl attachment to the N-terminal of VP0. The two mutants SVA-VP0-G1A/K-eGFP could not produce infectious virions even if we provided either additional L^Pro^ or wild-type VP0 (VP0-WT) to the BHK-21 cells (data not shown). These observations suggest that simply supplementing myristoylated VP0-WT could not fully replace the myristoylation-deficient VP0 (VP0-G1A/K) to form infectious virions. What factors caused such irreplaceability requires further investigation. These results suggest that exploring medicines targeting NMT might be an effective strategy for developing anti-virus drugs. While the majority of NMT1 substrates share a common amino acid sequence, studies have shown that proteins lacking a T or Ser residue at the fifth amino acid site could still be myristoylated [37,38]. Our findings showed that infectious virions could be rescued when the VP0 fifth-position T residue was replaced by A or K residue. Interestingly, the maximum viral titer of SVA-VP0-T5A or SVA-VP0-T5K decreased compared to SVA-WT (Figure 5B). This might be because the VP0 fifth-position T was more favorable to its myristoylation compared to A/K residue. This result indicates that substituting VP0 fifth-position T residue with A/K residue was an efficient method for weakening the virulence of SVA, which could be used in developing an SVA attenuated vaccine. 

In conclusion, SVA replication was regulated by NMT by catalyzing VP0 myristoylation, and VP0 myristoylation is indispensable for SVA replication. SVA replication could be adjusted by altering NMT activities or NMT1 quantity and substituting the VP0 fifth-position T residue with A/K residue.

## Data Availability

All the data available are included in the manuscript.

## Supplementary Materials

The following supporting information can be downloaded at https://www.mdpi.com/article/10.3390/pathogens13070601/s1, Figure S1: Myristoylation played a decisive role in determining VP0 subcellular localization. Table S1: Primers used in plasmid construction; Table S2: sgRNA used in BHK-NMT1-KO construction.
