# Supplementary material for: VP0 Myristoylation Is Essential for Senecavirus A Replication
Source: Pathogens. 2024 Jul 21;13(7):601. doi: 10.3390/pathogens13070601 (PMC11280471; doi:10.3390/pathogens13070601)
Supplement: Supplementary file 1 [file pathogens-13-00601-s001.zip › pathogens-3081288-supplementary.pdf]

Figure S1. Myristoylation played a decisive role in determining VP0 subcellular localization. pVP0-WT-eGFP was transfected into BHK-21 cells and BHK-NMT1-KO cells respectively. pVP0-G1A-eGFP was transfected into BHK-21 cells. After 24 h transfection, the subcellular localization of VP0-WT and VP0-G1A were observed under an inverted fluorescent microscope.

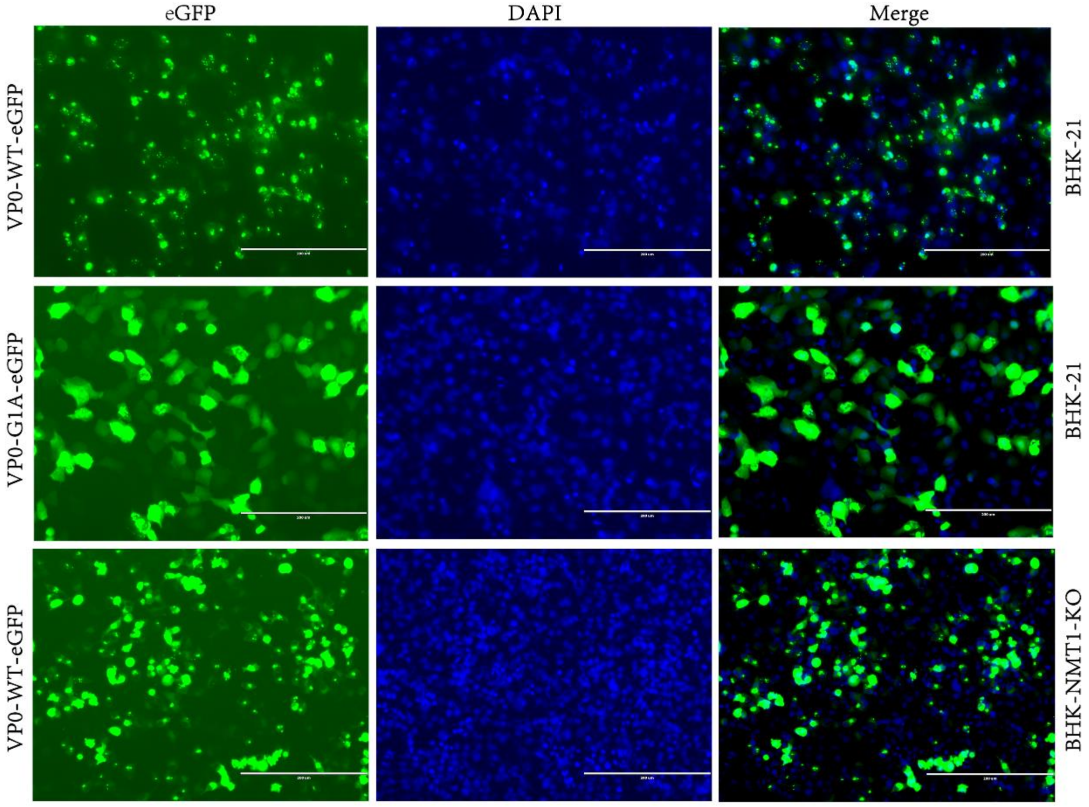

Table S1

Primers used in plasmid construction

Primer name	Primer sequence (5'-3')
NMT1-NheI-F	AGGCTAGCGCCACCATGGCGGATGAGAGTGAGACAGCAG
NMT1-NotI-R	AGCGGCCGCCTATTACTGTAACACCAACCCAACCTTC
VP0-NheI-F	TTAATACGACTCACTATAGGCTAGCGCCACCATGGGTAATGTTCAG ACAACCTCAAAG
VP0-linker-R	GCTGCCGCCTCTTTTGCGGCAGCTTCCTGTTCCTCGTCCGTCCCG GTCGTGAAAC
eGFP-linker-F	GAAGCTGCCGCAAAAGAGGCGGCAGCCAAGATGGTGAGCAAGG GCGAGGAGCTG
eGFP-NotI-R	TATCTTATCATGTCTGCTCGAAGCGGCCGCTTA CTTGTACAGCTCGTCCATGCCG
VP0-NheI-F	CTATAGGCTAGCACTAGTTAATAC
VP0-G1A-SacII-R	TTGCCGCGGGAATCAAAATCATTCTTTGAGGTTGTCTGAACATTG CCCTGTAGTTC

VP0-G1K-SacII-R	TTGCCGCGGGAATCAAAATCATTCTTTGAGGTTGTCTGAACATTCTTCTGTAGTTC
VP0-T5A-SacII-R	TTGCCGCGGGAATCAAAATCATTCTTTGAGGTGCCCTGAACATTACCCTGTAGTTC
VP0-T5K-SacII-R	TTGCCGCGGGAATCAAAATCATTCTTTGAGGTCTTCTGAACATTACCCTGTAGTTC

Table S2

sgRNA used in BHK-NMT1-KO construction

sgRNA name	oligos（5'-3'）
sgRNA-1	CACCGCTGCAGCGATTGCGAGAACG
sgRNA-2	CACCGCAATGACACAGATTCAGCCC
sgRNA-3	CACCGTCGCTGCCGCAGATGATGGA
